# Educational and health outcomes of children and adolescents receiving antidepressant medication: Scotland-wide retrospective record linkage cohort study of 766 237 schoolchildren

**DOI:** 10.1093/ije/dyaa002

**Published:** 2020-02-19

**Authors:** Michael Fleming, Catherine A Fitton, Markus F C Steiner, James S McLay, David Clark, Albert King, Daniel F Mackay, Jill P Pell

**Affiliations:** d1 Institute of Health and Wellbeing, University of Glasgow, Glasgow, UK; d2 Department of Child Health, University of Aberdeen, Aberdeen, UK; d3 Information Services Division, Edinburgh, UK; d4 ScotXed, Scottish Government, Edinburgh, UK

**Keywords:** Depression, educational outcomes, health, population cohort, record linkage, prescribing

## Abstract

**Background:**

Childhood depression is relatively common, under-researched and can impact social and cognitive function and self-esteem.

**Methods:**

Record linkage of routinely collected Scotland-wide administrative databases covering prescriptions [prescribing information system (PIS)], hospitalizations (Scottish Morbidity Records 01 and 04), maternity records (Scottish Morbidity Records 02), deaths (National Records of Scotland), annual pupil census, school absences/exclusions, special educational needs (Scottish Exchange of Educational Data; ScotXed), examinations (Scottish Qualifications Authority) and (un)employment (ScotXed) provided data on 766 237 children attending Scottish schools between 2009 and 2013 inclusively. We compared educational and health outcomes of children receiving antidepressant medication with their peers, adjusting for confounders (socio-demographic, maternity and comorbidity) and explored effect modifiers and mediators.

**Results:**

Compared with peers, children receiving antidepressants were more likely to be absent [adjusted incidence rate ratio (IRR) 1.90, 95% confidence interval (CI) 1.85–1.95] or excluded (adjusted IRR 1.48, 95% CI 1.29–1.69) from school, have special educational needs [adjusted odds ratio (OR) 1.77, 95% CI 1.65–1.90], have the lowest level of academic attainment (adjusted OR 3.00, 95% CI 2.51–3.58) and be unemployed after leaving school (adjusted OR 1.88, 95% CI 1.71–2.08). They had increased hospitalization [adjusted hazard ratio (HR) 2.07, 95% CI 1.98–2.18] and mortality (adjusted HR 2.73, 95% CI 1.73–4.29) over 5 years’ follow-up. Higher absenteeism partially explained poorer attainment and unemployment. Treatment with antidepressants was less common among boys than girls (0.5% vs 1.0%) but the associations with special educational need and unemployment were stronger in boys.

**Conclusions:**

Children receiving antidepressants fare worse than their peers across a wide range of education and health outcomes. Interventions to reduce absenteeism or mitigate its effects should be investigated.

**Key Messages**

- Children receiving antidepressants have poorer education and health outcomes compared with their peers.
- Compared with peers, children receiving antidepressants have increased hospitalization and mortality, more school absenteeism, greater school exclusion for challenging/disruptive behaviour, greater special educational need, poorer academic attainment and increased unemployment.
- Absenteeism partially explained poorer attainment and unemployment. Interventions to reduce absenteeism or mitigate its effects should be investigated.

## Background

Depression prevalence is estimated at 4.4% worldwide,^1^ 4.7% in Western Europe^2^ and 6.4–12.2% in the UK.^3^ Estimates vary through differing ascertainment methods^4–12^ and because depression may be undiagnosed^13^ or untreated. Worldwide, 2.6% of children and adolescents experience depressive disorders and 1.3% major depression.^14^ Prevalence is increasing,^15^ higher amongst girls^16–18^ and greater in older children, affecting 2.8% under 13 years of age and 5.8% of adolescents.^16^^,^^19^ Management includes antidepressant medication, cognitive–behavioural therapy and psychotherapy. Childhood antidepressant use varies between 0.2% and 1.6% and is 1.1% in the UK.^20–22^ Whilst antidepressants can treat several disorders, the most common prescribing reason is depression.^23–26^

Depression impacts cognition, social function and self-esteem,^5^^,^^6^^,^^27^ and therefore potentially school performance. Studies on school attendance are lacking and findings on academic achievement^4^^,^^5^^,^^7^^,^^27–30^ and further/higher education conflicting.^7^^,^^8^ Some suggest depressed children drop out of school earlier,^31^^,^^32^ whereas others do not,^8^ and results are inconsistent within studies.^9^ Data regarding all-cause hospitalization and mortality are sparse, but reports suggest depressed children suffer more violent, traffic-related and unintentional injuries,^33^ increased non-suicidal self-injury^34^ and suicide.^35^ Conflicting evidence may reflect different ascertainment methodologies including: self,^6–10^ parental,^7^^,^^11^ teacher^6^ and peer^12^ report, physician diagnosis,^5^ hospitalization^4^ and attempted suicide.^32^ Previous studies focused on a small number of outcomes, only two included >10 000 participants^11^^,^^28^ and some were limited by cross-sectional design.^4^^,^^5^^,^^12^^,^^30^

This study investigates outcomes for schoolchildren receiving antidepressants, regardless of indication, but with a focus on the medications most likely to be prescribed for depression. To our knowledge, it is the first countrywide cohort study to compare a wide range of educational and health outcomes between schoolchildren receiving antidepressants and peers. We questioned whether, compared with peers, children receiving antidepressants: (i) have increased school absenteeism; (ii) have increased school exclusion; (iii) have greater special educational need (SEN); (iv) have poorer academic attainment; (v) leave school earlier; (vi) have higher unemployment; (vii) have increased all-cause hospital admissions; (viii) have increased hospitalization for injury, poisoning or trauma; and (ix) have greater mortality. We hypothesized that children receiving antidepressants perform more poorly than peers across all outcomes.

## Methods

### Databases

We linked Scotland-wide individual-level data from five health and four education databases, held respectively by the Information Services Division (ISD) of the National Health Service and the Scottish Exchange of Educational Data (ScotXed) described previously.^36–41^ The prescribing information system (PIS) collects information from prescriptions dispensed to Scottish residents by community pharmacies or primary care. The Scottish Morbidity Record (SMR) 02 maternity database collects data pertaining to mother and baby for all births in Scotland. SMR01 and SMR04 record acute and psychiatric hospital admissions, including admission and discharge dates and International Classification of Diseases (ICD-10) diagnostic codes. National Records of Scotland collect death certificates, including date and ICD-10 cause of death.

The pupil census, conducted annually by all Scottish local-authority-run primary, secondary and special schools, records demographic information and instances of SEN including type. Absences and exclusions, collected prospectively, are appended at the end of each year. The Scottish Qualifications Authority collects exam results for schoolchildren sitting exams in the last 3 years of high school (S4–S6). The school-leaver database records pupil whereabouts 3–6 months after leaving school: paid/voluntary employment, higher/further education, training or unemployment.

### Inclusion criteria, definitions and outcomes

We included records for children appearing on at least one pupil census between 2009 and 2013. The mean years of school attendance during the study was 3.65 (range 1–5). We excluded children aged <4 years or >19 years and restricted the study to singletons. Using PIS data, we identified children prescribed at least one antidepressant during the study period: any tricyclic antidepressant, selective serotonin reuptake inhibitor (SSRI) or the serotonin norepinephrine reuptake inhibitors mirtazapine or venlafaxine. Children not prescribed an antidepressant listed above but prescribed alternative antidepressants from the British National Formulary (BNF) chapter 4.3 were excluded from the study (Supplementary Figure 1, available as Supplementary data at *IJE* online). Children not prescribed any antidepressants comprised the peer group.

We studied six educational outcomes. (i) number of days absent; (ii) number of exclusions (suspensions or expulsions) for challenging/disruptive behaviour; and (iii) records of SEN were recorded annually for every child and analysed yearly. Absence and exclusion data were available for 2009, 2010 and 2012. (iv) summarized academic achievement; (v) percentage of children leaving school before 16 years of age; and (vi) subsequent unemployment, were derived and analysed as single, end-of-school outcomes.

SEN comprised intellectual disabilities, dyslexia, learning difficulties physical/motor/sensory impairment, language/speech disorder, autistic spectrum disorder, physical/mental health problems and social/emotional/behavioural difficulties. Academic achievement (low, basic, broad general or high) was derived using total exam awards attained at each level of the Scottish Credit Qualifications Framework (SCQF) over the last 3 years of high school (S4–S6).^37^^,^^42^ Leaver destination was collapsed into education/employment/training or unemployment. Children were followed on commencement of antidepressant treatment (exposed) or their first school year (non-exposed) for three health outcomes: (vii) all-cause hospitalization; (viii) hospitalization for injury, poisoning or trauma (primary ICD-10 codes S00-T98), including intentional self-harm (secondary ICD-10 codes X60–X84); and (ix) death. Hospitalizations and deaths were available until September 2014, providing a maximum of 5 years’ follow-up.

We adjusted for socio-demographic confounders. The pupil census provided child’s age, gender and ethnicity. Area socio-economic deprivation was derived from postcode of residence using the Scottish Index of Multiple Deprivation (SIMD) 2012 and children were allocated to general-population quintiles. We also adjusted for maternal and obstetric confounders, previously associated with SEN.^43–45^ Linkage to SMR02 provided maternal age at delivery, parity, maternal smoking, gestation at delivery, mode of delivery and 5-minute Apgar score, and we derived sex- and gestation-specific birthweight centiles. Finally, using PIS to identify children treated for attention deficit hyperactivity disorder (ADHD),^37^^,^^38^ epilepsy,^37^^,^^39^ diabetes^37^^,^^40^ and asthma,^37^^,^^41^ we adjusted for chronic conditions independently associated with poor educational and health outcomes that can coexist alongside depression.^37–41^

### Statistical analyses

Characteristics of children on antidepressants were compared with peers using chi-square tests (categorical data) and chi-square tests for trend (ordinal data). SEN, absences and exclusions were analysed as yearly outcomes using longitudinal generalized estimating equations (GEEs) adjusting for correlated observations on the same pupil across different years.^46^ The exposure, prescribed antidepressants over the same school year as the outcome, predated each outcome. The user-written quasi-likelihood under the independence model criterion (QIC) statistic^47^ compared different correlation structures with the lowest trace QIC deemed most appropriate. SEN was modelled using GEE analyses with a binomial distribution and logit link to produce odds ratios (ORs). Numbers of days absent and number of exclusions were modelled using GEE analyses with a negative binomial distribution and log link to produce incidence rate ratios (IRRs). The number of possible yearly attendances was an offset variable adjusting for individual exposure time in the latter two outcomes.

Age at leaving school, subsequent unemployment, final academic attainment, hospitalization and death were analysed as one-off outcomes using summarized data per pupil. Longitudinal methods were not required and the exposure, ever prescribed antidepressants during the study period, predated each outcome. Age at leaving school (binomial), subsequent unemployment (binomial) and final academic attainment (generalized ordinal) were analysed using logistic regression to produce ORs. Hospitalization and death were modelled using time-to-event analyses: Cox proportional hazards to produce hazard ratios (HRs) or Poisson piecewise regression to produce IRRs where the proportionality assumption of the Cox model was not met. In the time-to-event models, children prescribed antidepressants were followed from their treatment-commencement date within the study period. The pupil census is recorded each September, shortly after school term commences. Children not prescribed antidepressants in the study period were followed from their earliest pupil census date, as previously described.^37^

All models were adjusted for socio-demographic and maternity factors and co-morbid conditions. Model 1 was unadjusted; Model 2 adjusted for age, sex, deprivation quintile, ethnic group, maternal age, maternal smoking, parity, mode of delivery, gestation at delivery, sex- and gestation-specific birthweight centile and 5-minute Apgar score; and Model 3 additionally adjusted for ADHD, epilepsy, asthma and diabetes. We explored age, sex and deprivation as effect modifiers by testing for statistical interactions and undertaking subgroup analyses where interactions were significant. We reanalysed the attainment and unemployment models adjusting for absenteeism to explore whether it was a mediator of either or both. We also reanalysed unemployment adjusting for attainment to uncover any mediating effect. We reran the original attainment and unemployment models excluding children with SEN. Finally we reran the main analyses using a new exposed group, to compare children receiving fluoxetine, citalopram or either with those receiving no antidepressants. Children receiving antidepressants excluding fluoxetine or citalopram were excluded from the analyses. All analyses were undertaken using Stata MP version 14.1. Supplementary Table 1, available as Supplementary data at *IJE* online, summarizes the type and frequency of each outcome variable, corresponding denominator populations, length of follow-up and analytical methods employed.

## Results

Between 2009 and 2013, 766 244 singleton children attended Scottish schools and contributed 2 793 157 pupil records. The mean school years contributed per pupil was 3.65 (range 1–5). Of 766 237 children eligible for inclusion, 5342 (0.7%) received antidepressants during the study period: 1752 (0.5%) boys and 3590 (1.0%) girls. Antidepressants were dispensed in 20 328 of the 2 793 157 pupil school years analysed (0.7%). Children receiving antidepressants were older on average than peers, less likely to live in the most deprived quintile , smaller for gestational age at birth and their mothers were more likely to have smoked during pregnancy. They were more likely to receive medication for diabetes, ADHD, epilepsy and asthma (Table 1). The percentage of data missing within each variable was <0.2% excluding parity (0.6%), Apgar score (1.1%), ethnicity (1.8%) and smoking during pregnancy (9.9%) (Table 1). Missing values for the latter two were analysed as ‘unknown’.

**Table 1.** Characteristics of schoolchildren by receipt of antidepressant medication

		No antidepressants		Antidepressants		
		*N* = 760 895		*N* = 5342		
		*N*	%	*N*	%	*P-*value
Socio-demographic factors (recorded annually on pupil census)						
Sex						
	Male	388 537	51.1	1752	32.8	<0.001
	Female	372 358	48.9	3590	67.2	
	Missing	0		0		
Average age over all school years attended						
	Mean (SD)	10.92 (3.65)		14.00 (2.47)		<0.001
Deprivation quintile^a^						
	1 (most deprived)	172 776	22.7	1016	19.1	<0.001
	2	152 464	20.1	1102	20.7	
	3	146 776	19.3	1147	21.5	
	4	148 445	19.5	1077	20.2	
	5 (least deprived)	139 849	18.4	991	18.6	
	Missing	585		9		
Ethnic group						
	White	722 929	96.2	5180	97.7	<0.001
	Asian	17 715	2.4	62	1.2	
	Black	1963	0.3	2	0.0	
	Mixed	6684	0.9	44	0.8	
	Other	2064	0.3	12	0.2	
	Missing	9540		42		
Medication prescribed for other conditions during study period						
	Diabetes	3271	0.4	59	1.1	<0.001
	Asthma	45 312	6.0	587	11.0	<0.001
	Epilepsy	4857	0.6	454	8.5	<0.001
	ADHD	7222	0.9	191	3.6	<0.001
Maternity factors (recorded at time of birth)						
Maternal age (years)						
	≤24	208 448	27.4	1430	26.8	0.015
	25–29	222 830	29.3	1705	31.9	
	30–34	215 418	28.3	1515	28.4	
	≥35	114 187	15.0	692	13.0	
	Missing	12		0		
Maternal smoking						
	No	487 887	72.4	3223	69.3	<0.001
	Yes	186 356	27.6	1430	30.7	
	Missing	86 652		689		
Parity						
	0	343 259	45.3	2404	45.1	0.648
	1	262 234	34.6	1905	35.7	
	>1	151 541	20.0	1027	19.2	
	Missing	3861		6		
Mode of delivery						
	SVD	512 522	67.4	3692	69.1	0.001
	Assisted vaginal	91 041	12.0	616	11.5	
	Breech vaginal	2214	0.3	19	0.4	
	Elective CS	57 912	7.6	402	7.5	
	Emergency CS	97 041	12.8	613	11.5	
	Other	163	0.0	0	0.0	
	Missing	2		0		
Gestation (weeks)						
	<28	1143	0.1	11	0.2	0.013
	28–32	6995	0.9	63	1.2	
	33–36	35 346	4.6	255	4.8	
	37	37 346	4.9	273	5.1	
	38	95 288	12.5	702	13.2	
	39	157 658	20.7	1080	20.2	
	40	228 780	30.1	1649	30.9	
	41	170 093	22.4	1099	20.6	
	42	26 926	3.5	198	3.7	
	>42	762	0.1	8	0.1	
	Missing	558		4		
Sex-gestation-specific birthweight centile						
	1–3	31 253	4.1	232	4.3	0.003
	4–10	68 129	9.0	517	9.7	
	11–20	90 638	11.9	710	13.3	
	21–80	447 064	58.8	3054	57.2	
	81–90	64 925	8.5	437	8.2	
	91–97	40 949	5.4	270	5.1	
	98–100	16 963	2.2	116	2.2	
	Missing	974		6		
5-minute Apgar						
	1–3	3674	0.5	35	0.7	0.180
	4–6	7252	1.0	50	0.9	
	7–10	742 161	98.5	5244	98.4	
	Missing	7808		13		

ADHD, attention deficit hyperactivity disorder; *N*, number; SVD, spontaneous vaginal delivery; CS, Caesarean section. *P*-values created using chi-square tests for categorical data, chi-square tests for trend for ordinal data and *t*-tests for continuous data (age).

a Deprivation quintile can change across different school years if a child’s family move house. Therefore, the most commonly occurring deprivation quintile was chosen for each pupil across all of their school records in the study period. If two or more deprivation quintiles occurred equally, then the last known deprivation quintile was used in the analyses.

Antidepressant use varied by gender and age. Among treated children, 67.2% were girls, 32.8% were boys, 18.0% commenced treatment at under 11 years of age, 44.9% aged 11–14 years and 37.1% over 14 years of age (Supplementary Table 2, available as Supplementary data at *IJE* online). Fluoxetine (41.1%), amitriptyline (31.5%), sertraline (15.4%) and citalopram (11.9%) were the most commonly prescribed medications and SSRIs (61.7%) the most common drug class. Treatment over the period was stable; 89.1% of children on antidepressants received one drug type and 96.9% one drug class (Supplementary Table 2, available as Supplementary data at *IJE* online).

Analyses of absences and exclusions included 1 597 379 pupil records for 702 203 children. Children on antidepressants had more annual absences (median 15.5 vs 7.5 days among peers) evident in Model 1 [IRR 2.23, 95% confidence interval (CI) 2.17–2.29], Model 2 (IRR 1.95, 95% CI 1.90–2.00) and Model 3 (IRR 1.90, 95% CI 1.85–1.95). The association strengthened with age: IRR 2.01 (95% CI 1.94–2.07) >14 years of age compared with IRR 1.56 (95% CI 1.45–1.68) <11 years (interaction, *p* < 0.001). The association weakened with increasing deprivation (interaction, *p* < 0.001): IRR 2.37 (95% CI 2.21–2.53) in the least-deprived quintile compared with IRR 1.56 (95% CI 1.48–1.66) in the most. However, this was due to higher baseline absenteeism among children not on antidepressants in deprived areas.

Children on antidepressants were more likely to be excluded in Model 1 (IRR 1.86, 95% CI 1.63–2.13), Model 2 (IRR 1.65, 95% CI 1.45–1.88) and Model 3 (IRR 1.48, 95% CI 1.29–1.69); 7.3% were excluded from school at least once during the study period compared with 3.8% of peers. The association was stronger in younger children; IRR 2.11 (95% CI 1.36–3.27) <11 years of age compared with IRR 1.38 (95% CI 1.16–1.65) >14 years (interaction, *p* < 0.001).

A greater percentage of children on antidepressants had a SEN compared with peers (27.4% vs 15.1%). Associations with SEN were stronger in boys than girls and increased with decreasing deprivation (interactions, *p* < 0.001) based on analyses of 2 793 157 pupil records pertaining to 766 237 children (Table 2).

**Table 2.** Association between receipt of antidepressants and record of special educational need: overall and by sex, age^d^ and area deprivation

	Model 1		Model 2		Model 3	
	*N* = 2 793 157 (766 237)		*N* = 2 741 516 (753 133)		*N* = 2 741 516 (753 133)	
	OR	95% CI	OR	95% CI	OR	95% CI
Overall	1.99	1.87–2.12	2.24	2.10–2.39	1.77	1.65–1.90
Boys^a^	2.71	2.47–2.97	2.66	2.42–2.94	2.06	1.85–2.30
Girls^a^	2.02	1.86–2.20	1.93	1.77–2.11	1.54	1.40–1.70
<11 years^b^	2.74	2.36–3.19	2.73	2.32–3.20	2.01	1.68–2.41
11–14 years^b^	1.52	1.39–1.67	1.78	1.62–1.96	1.42	1.28–1.58
>14 years^b^	2.00	1.86–2.15	2.46	2.29–2.65	1.99	1.84–2.16
1 (more deprived)^c^	1.60	1.40–1.83	1.73	1.51–1.98	1.36	1.17–1.58
2^c^	1.86	1.63–2.13	2.05	1.79–2.35	1.62	1.39–1.88
3^c^	1.94	1.70–2.21	2.19	1.91–2.50	1.73	1.49–2.01
4^c^	2.40	2.09–2.75	2.72	2.37–3.13	2.15	1.84–2.51
5 (least deprived)^c^	2.70	2.32–3.13	2.93	2.51–3.42	2.32	1.96–2.74

Model 1—unadjusted.

Model 2—adjusted for age at outcome, sex, deprivation quintile, ethnic group, maternal age, maternal smoking, parity, mode of delivery, gestation at delivery, sex- and gestation-specific birthweight centile and 5-minute Apgar score.

Model 3—also adjusted for co-morbid conditions (diabetes, asthma, epilepsy and attention deficit hyperactivity disorder).

*N*—number of records (number of children).

a Subgroups therefore not adjusted for sex.

b Subgroups therefore not adjusted for age.

c Subgroups therefore not adjusted for deprivation quintile.

d Age—age at receiving special educational need.

OR, odds ratio; CI, confidence interval.

All *p* < 0.001.

On analysing exam grades for 139 199 children, 2340 (1.7%) received antidepressants. The percentage obtaining the lowest level of academic attainment was greater among children on antidepressants (7.6%) than peers (4.6%). They were more likely to attain the lowest level of attainment in Model 1 (OR 1.69, 95% CI 1.45–1.97), Model 2 (OR 3.44, 95% CI 2.89–4.09) and Model 3 (OR 3.00, 95% CI 2.51–3.58). The relative impact was lower in the most deprived children (OR 3.09, 95% CI 2.22–4.29) than in the least deprived (OR 5.72, 95% CI 3.37–9.70) (interaction, *p* < 0.001), due to the higher absolute risk among unaffected children in deprived areas. Adjustment for absenteeism attenuated the association (fully adjusted OR 1.65, 95% CI 1.35–2.02). The original association remained after excluding children with SEN (fully adjusted OR 2.99, 95% CI 2.38–3.77).

**Figure 1.**
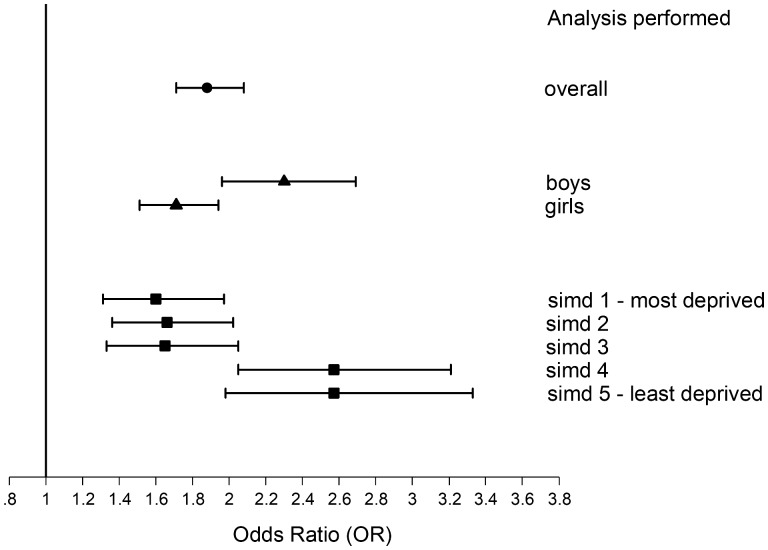
Forest plot of the associations between antidepressant treatment and unemployment among 217 919 schoolchildren: overall and by sex and area deprivation. Adjusted for age, sex, deprivation quintile and ethnic group, maternal age, maternal smoking, parity, mode of delivery, gestation at delivery, sex- and gestation-specific birthweight centile, 5-minute Apgar score and co-morbid chronic conditions (attention deficit hyperactivity disorder, epilepsy, asthma, diabetes). SIMD, Scottish Index of Multiple Deprivation. Solid circles/triangles/squares denote odds ratios; bars denote 95% confidence intervals.

Of 217 919 school-leavers, 3394 (1.6%) received antidepressants. Quitting school before 16 years of age occurred less among children on antidepressants (26.7%) than peers (28.8%). However, the association disappeared after adjusting for confounders (OR 0.98, 95% CI 0.90–1.06). Unemployment was higher among children on antidepressants compared with peers (16.3% vs 10.3%). They were more likely to be unemployed 6 months post school in Model 1 (OR 1.69, 95% CI 1.54–1.85), Model 2 (OR 1.98, 95% CI 1.80–2.18) and Model 3 (OR 1.88, 95% CI 1.71–2.08). The association with unemployment was stronger in boys (OR 2.30, 95% CI 1.96–2.69) than in girls (OR 1.71, 95% CI 1.51–1.94) (interaction, *p* = 0.001) (Figure 1). The relative association was also stronger in the least (OR 2.57, 95% CI 1.98–3.33) than the most (OR 1.60, 95% CI 1.31–1.97) deprived quintiles (interaction, *p* < 0.001), again reflecting underlying absolute risk. Associations with unemployment remained after excluding children with SEN (fully adjusted OR 1.97, 95% CI 1.76–2.21). Adjusting the original model for absenteeism (fully adjusted OR 1.36, 95% CI 1.23–1.51) and then adding attainment (fully adjusted OR 1.30, 95% CI 1.13–1.49) also attenuated the original association.

**Figure 2.**
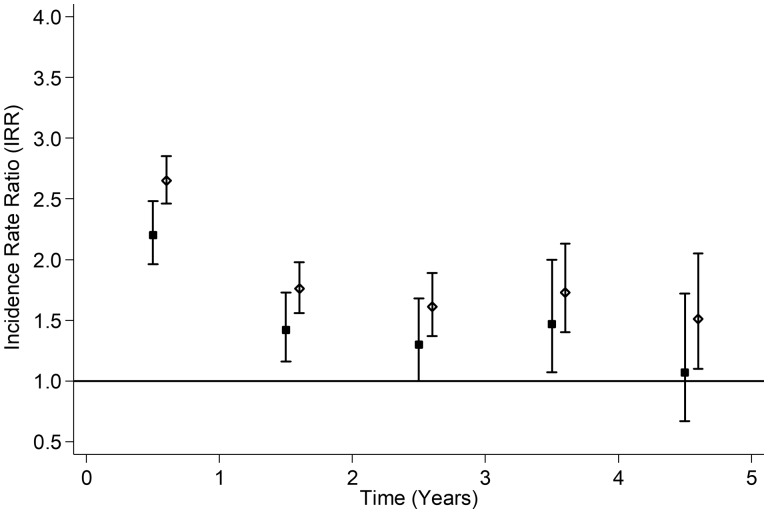
Forest plot of the association between antidepressant treatment and all-cause hospitalization among 766 237 schoolchildren by time from commencement of treatment and by sex. Adjusted for age, deprivation quintile and ethnic group, maternal age, maternal smoking, parity, mode of delivery, gestation at delivery, sex- and gestation-specific birthweight centile, 5-minute Apgar score and co-morbid chronic conditions (attention deficit hyperactivity disorder, epilepsy, asthma, diabetes). SIMD, Scottish Index of Multiple Deprivation. Boys = solid square; girls = hollow diamond; bars denote 95% confidence intervals.

**Figure 3.**
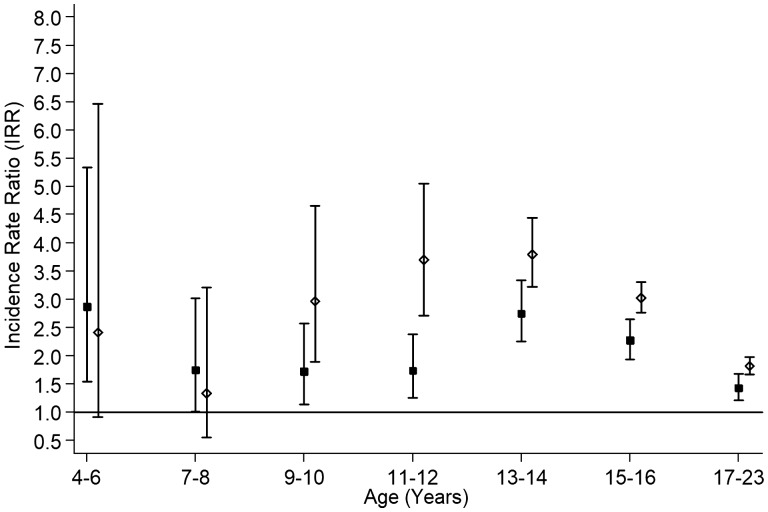
Forest plot of the association between antidepressant treatment and all-cause hospitalization among 766 237 schoolchildren by age at first admission in follow-up and by sex. Adjusted for age, deprivation quintile and ethnic group, maternal age, maternal smoking, parity, mode of delivery, gestation at delivery, sex- and gestation-specific birthweight centile, 5-minute Apgar score and co-morbid chronic conditions (attention deficit hyperactivity disorder, epilepsy, asthma, diabetes). SIMD, Scottish Index of Multiple Deprivation. Boys = solid square; girls = hollow diamond; bars denote 95% confidence interval.

Over 4.33 years’ follow-up (range 1–5 years), 157 291 (20.5%) of 766 237 children were hospitalized. More children receiving antidepressants were hospitalized (34.2%) than peers (20.4%). A Cox regression model reflected this (fully adjusted HR 2.07, 95% CI 1.98–2.18) although proportional hazards were not met (*p* < 0.001). Therefore, Poisson piecewise regression models, stratified by sex, were run by period of follow-up (Figure 2) and age of child at hospital admission (Figure 3). Children on antidepressants had an elevated risk of hospitalization throughout, particularly in the first year of follow-up (Figure 2) and between 11 and 16 years of age (Figure 3). The association was stronger in girls than in boys (Figures 2 and 3). Injury, poisoning and trauma accounted for 27.8% of hospitalizations among children on antidepressants compared with 21.3% among peers. Further, 9.5% of children on antidepressants had at least one admission for injury, poisoning or trauma compared with 4.3% of peers. Amongst children on antidepressants admitted for injury, poisoning or trauma, 66.6% were for intentional self-harm. The corresponding percentage among peers was 10.0%. The average age for all first injury, poisoning or trauma admissions was 12.82 years (SD = 4.40) but higher for intentional self-harm [16.53 years (SD = 2.07)] than for unintentional injury [12.36 years (SD = 4.40)]. Over follow-up, 491 children (22 on antidepressants and 469 peers) among 766 237 children attending school between 2009 and 2013 died. Risk of death was higher among children on antidepressants in Model 1 (HR 5.76, 95% CI 3.74–8.88), Model 2 (HR 6.25, 95% CI 4.05–9.65) and Model 3 (HR 2.73, 95% CI 1.73–4.29). When the main models were rerun using the more stringent definition of antidepressant medication, the associations persisted and were generally greater in magnitude (Table 3).

**Table 3.** Comparison of results based on narrow and wide definitions of antidepressant medication

		Fluoxetine		Citalopram		Fluoxetine or Citalopram		Any antidepressant	
		*N* = 766 237 (2200)		*N* = 766 237 (638)		*N* = 766 237 (2692)		*N* = 766 237 (5342)	
		Effect size	95% CI	Effect size	95% CI	Effect size	95% CI	Effect size	95% CI
Absence^g^		2.18	2.09–2.27	2.03	1.88–2.19	2.14	2.06–2.21	1.90	1.85–1.95
Exclusion for disruptive behaviour^g^		1.51	1.26–1.81	1.47^a^	1.05–2.06	1.55	1.32–1.82	1.48	1.29–1.69
SEN^h^		2.13	1.92–2.36	1.57	1.28–1.94	2.02	1.84–2.23	1.77	1.65–1.90
Attainment^i^	General/basic/low	2.24	1.91–2.61	2.34	1.87–2.92	2.26	1.97–2.60	1.86	1.68–2.05
	Basic/low	2.75	2.34–3.24	2.34	1.87–2.92	2.74	2.37–3.17	1.99	1.78–2.23
	Low	4.44	3.52–5.60	2.34	1.87–2.92	3.83	3.07–4.77	3.00	2.51–3.58
	High (reference)	1.00		1.00		1.00		1.00	
Left school before 16 years of age^j^		1.25	1.11–1.41	0.72^b^	0.58–0.91	1.11^c^	1.00–1.24	0.98^d^	0.90–1.06
Unemployment^j^		2.60	2.28–2.97	2.21	1.73–2.83	2.48	2.20–2.80	1.88	1.71–2.08
Admission^k^		2.50	2.33–2.69	2.16	1.89–2.46	2.41	2.26–2.57	2.07	1.98–2.18
Mortality^k^		3.21^e^	1.56–6.58	1.43^f^	0.20–10.22	3.00^e^	1.52–5.91	2.73	1.73–4.29

* Adjusted for age, sex, deprivation quintile, ethnic group, maternal age, maternal smoking, parity, mode of delivery, gestation at delivery, sex- and gestation-specific birthweight centile, 5-minute Apgar score and co-morbid conditions (diabetes, asthma, epilepsy and attention deficit hyperactivity disorder).

*N*—total number of children (total number of children on medication).

OR, odds ratio; CI, confidence interval.

All *p* < 0.001 with the exception of: ^a^*p* < 0.05; ^b^*p* = 0.005; ^c^*p* = 0.051; ^d^*p* = 0.559; ^e^*p* = 0.001; ^f^*p* = 0.722.

g 1 597 379 records (702 203 pupils) analysed using Generalised Estimating Equations with a negative binomial distribution and log link function to produce incidence rate ratios (IRRs).

h 2 793 157 records (766 237 pupils) analysed using Generalised Estimating Equations with a binomial distribution and logit link function to produce odds ratios (ORs).

i 139 199 pupils analysed using generalized ordinal logistic regression to produce ORs.

j 217 919 pupils analysed using binomial logistic regression to produce ORs.

k 766 237 pupils analysed using Cox regression to produce hazard ratios (HRs).

## Discussion

Children on antidepressants fared worse than peers across various outcomes: more school absences and exclusions; greater SEN and unemployment; poorer examination results; and excess hospitalization and death. Poorer attainment and higher unemployment were partially explained by increased absenteeism.

Gender differences in depression prevalence vary with age. Within our study period, commencement of antidepressants in girls was comparable to boys below 11 years of age, but two-fold higher in older age groups. Previous studies report lower depression prevalence in girls before 13–14 years of age, but double that of boys above this age.^31^^,^^48^ Reasons include under-diagnosis and under-treatment among boys,^31^ more biological and social challenges for girls entering adolescence^49^ and poorer coping mechanisms among girls.^48^

In our study, antidepressants were associated with worse educational outcomes in boys. Depressive symptoms, such as self-criticism and helplessness, are inconsistent with society’s expectations of male behaviour; therefore, parents, teachers and peers may support boys less.^50^ In contrast, girls on antidepressants were more likely to be hospitalized. Further research should determine whether this reflects worse health or a greater willingness to seek medical help. Previous studies reported gender differences in academic performance, some reporting stronger associations in boys^11^^,^^51^ and others in girls.^6–9^^,^^52^^,^^53^

Depressed children often present with irritability, restlessness, aggression and hyperactivity, especially in early childhood.^54^ Dominant symptoms in adolescence are suicidal thoughts, hopelessness, social isolation, drug or alcohol use, overeating, oversleeping and rage.^13^ This could explain the stronger association with school exclusion in younger children and the stronger association with absence in adolescence.

Receipt of antidepressants is a reasonable proxy of depression, more objective and less prone to bias, than self, parental or teacher reports, but cannot differentiate disease and medication effects, may only identify children with severe symptoms and may be incomplete due to misdiagnosis or under-treatment, especially in boys.^13^ Additionally, we only had prescribing data from 2009 onwards. Nevertheless, ascertainment of cases using school, not health, records ensured non-restriction to severe hospitalized depression. Antidepressants are not required for all cases of childhood depression; some are used for conditions such as anxiety, obsessive compulsive disorder or enuresis. Without primary-care records, we could not confirm the clinical indications for medication. However, previous studies report that depression is the main reason for prescribing SSRI antidepressants.^23–26^ We partially addressed this limitation by repeating our analyses including only children receiving fluoxetine (the recommended treatment and only drug licensed in the UK for treating depression in children under 16 years of age) and citalopram (the most common second-line treatment). Whilst these can be prescribed for other indications, a previous study demonstrated that 62.4% and 62.2% of children prescribed fluoxetine and citalopram were depressed.^24^ Associations remained after running these sensitivity analyses and were stronger than previously observed, suggesting our initial findings were unlikely to have been due to misclassification.

Ours was a large, non-selective study covering the whole of Scotland. The relationship between depression and academic performance can be bidirectional,^6^ challenging the robustness of previous cross-sectional studies. A cohort approach ensured antidepressant use predated education and health outcomes. We adjusted for several confounders; however, residual confounding is possible in any observational study. Consistent with previous studies, schoolchildren taking antidepressants had more co-morbid conditions. By including treatment of ADHD, epilepsy, diabetes and asthma as covariates, we demonstrated that adverse outcomes were independently associated with antidepressant treatment, not merely coexisting conditions. The large study population provided sufficient power to test for interactions and conduct subgroup analyses, and we analysed a wide range of outcomes in the same cohort. Missing data across all covariates did not exceed 1.9% and, for most analyses, were considerably lower. Given the sample size, we do not believe this affected the results and therefore did not impute data or run sensitivity analyses.

The study included only local-authority-maintained schools. However, in Scotland, only 5% of children attend private schools. In the 2011 Scottish Census, 11% of Scottish residents aged 5–19 years were born outside of Scotland, consistent with the 12% of schoolchildren we could not link to maternity records. Prevalence of antidepressant treatment was 0.7% among linked and unlinked pupils, suggesting bias was unlikely. The study used administrative databases established for other purposes that undergo regular quality assurance. Education and health records were linked using probabilistic matching, validated as 99% accurate for singletons.^36^

## Conclusion

Children with mental health conditions severe enough to require antidepressants fare poorly across a range of educational and health outcomes. In boys, antidepressant use was less common but associated with worse outcomes. Affected children should be identified early and supported to reduce the risk of school absence or exclusion and minimize longer-term impacts on employment and health.

## Supplementary Material

dyaa002_Supplementary_Data
