# Supplementary material for: Educational and health outcomes of children and adolescents receiving antidepressant medication: Scotland-wide retrospective record linkage cohort study of 766 237 schoolchildren
Source: Int J Epidemiol. 2020 Feb 19;49(4):1380–91. doi: 10.1093/ije/dyaa002 (PMC7660154; doi:10.1093/ije/dyaa002)
Supplement: dyaa002_Supplementary_Data [file dyaa002_supplementary_data.zip › dyaa002-suppl_data/ije-2018-07-0861-File008.docx]

Supplementary Table 1

| **Outcome** | **outcome measure** | **frequency outcome recorded** | **denominator** | **exposure – outcome time lag** | **Method of analysis** | **Measure** |
| --- | --- | --- | --- | --- | --- | --- |
| Absence | count | Annually* | 1,597,397 school records | same year | Longitudinal Generalised Estimating Equation with negative binomial distribution and log link | Incidence Rate Ratio |
| Exclusion | count | Annually* | 1,597,397 school records | same year | Longitudinal Generalised Estimating Equation with negative binomial distribution and log link | Incidence Rate Ratio |
| Special educational need | binary | Annually* | 2,793,157 school records | same year | Longitudinal Generalised Estimating Equation with binomial distribution and logit link | Odds Ratio |
| Attainment | Ordinal | Once | 139,199 pupils | range 1-5 years  mean 3.07 years | Ordinal Logistic Regression | Odds Ratio |
| left school <16 years of age | Binary | Once | 217,919 pupils | range 1-5 years  mean 2.57 years | Binary Logistic Regression | Odds Ratio |
| unemployed | Binary | Once | 217,919 pupils | range 1-5 years  mean 2.57 years | Binary Logistic Regression | Odds Ratio |
| Hospital admission (first) | Binary | Once | 766,237 pupils | range 1-5 years  mean 4.33 years | Cox proportional hazard model  Poisson piecewise regression | Hazard Ratio  Incidence Rate Ratio |
| Death | Binary | Once | 766,237 pupils | range 1-5 years  mean 4.33 years | Cox proportional hazard ratio Poisson piecewise regression | Hazard Ratio  Incidence Rate Ratio |

*number of outcomes measured per pupil: range 1-5; mean 3.7
