# Supplementary material for: Educational and health outcomes of children and adolescents receiving antidepressant medication: Scotland-wide retrospective record linkage cohort study of 766 237 schoolchildren
Source: Int J Epidemiol. 2020 Feb 19;49(4):1380–91. doi: 10.1093/ije/dyaa002 (PMC7660154; doi:10.1093/ije/dyaa002)
Supplement: dyaa002_Supplementary_Data [file dyaa002_supplementary_data.zip › dyaa002-suppl_data/ije-2018-07-0861-File009.docx]

**Supplementary Table 2.** Characteristics of schoolchildren on antidepressant medication

|  |  | **Children on antidepressants** | |
| --- | --- | --- | --- |
|  |  | N=5,342 | |
|  |  | N | % |
| **Sex** |  |  |  |
|  | Male | 1,752 | 32.8 |
|  | Female | 3,590 | 67.2 |
| **Age at first antidepressant prescription (years)** | |  |  |
|  | <10 | 960 | 18.0 |
|  | 10-14 | 2,400 | 44.9 |
|  | >14 | 1,982 | 37.1 |
| **Mean (SD) age at first antidepressant prescription** | | |  |
|  |  | 13.0 (2.6) |  |
| **Antidepressant drug** | | |  |
|  | Fluoxetine | 2,201 | 41.1 |
|  | Amitriptyline | 1,686 | 31.5 |
|  | Sertraline | 823 | 15.4 |
|  | Citalopram | 639 | 11.9 |
|  | Imipramine | 274 | 5.1 |
|  | Mirtazapine | 91 | 1.7 |
|  | Nortriptyline | 92 | 1.7 |
|  | Escitalopram | 38 | 0.7 |
|  | Dusolepine | 32 | 0.6 |
|  | Trazodone | 28 | 0.5 |
|  | Clomipramine | 22 | 0.4 |
|  | Venlafaxine | 22 | 0.4 |
|  | Duloxetine | 14 | 0.3 |
|  | Paroxetine | 17 | 0.3 |
|  | Lofepramine | 12 | 0.2 |
|  | Other | 20 | 0.3 |
| **Antidepressant drug class** | | | |
|  | SSRI | 3,303 | 61.7 |
|  | Tricyclic | 2,096 | 39.2 |
|  | SNRI | 128 | 2.4 |
| **Number of different antidepressants prescribed during study** | | | |
|  | 1 | 4,771 | 89.1 |
|  | 2 | 562 | 10.5 |
|  | >=3 | 19 | 0.3 |
| **Number of different antidepressant drug classes prescribed during study** | | | |
|  | 1 | 5,184 | 96.9 |
|  | >=2 | 168 | 3.1 |
|  |  |  |  |

SSRI Selective Serotonin Reuptake Inhibitor; SNRI Selective Norepinephrine Reuptake Inhibitor

Other = Doxepin, Flupentixol, Fluvoxamine, Trimipramine, Tranylcypromine or Tryptophan
