# Supplementary figures and images for: Educational and health outcomes of children and adolescents receiving antidepressant medication: Scotland-wide retrospective record linkage cohort study of 766 237 schoolchildren
Source: Int J Epidemiol. 2020 Feb 19;49(4):1380–91. doi: 10.1093/ije/dyaa002 (PMC7660154; doi:10.1093/ije/dyaa002)

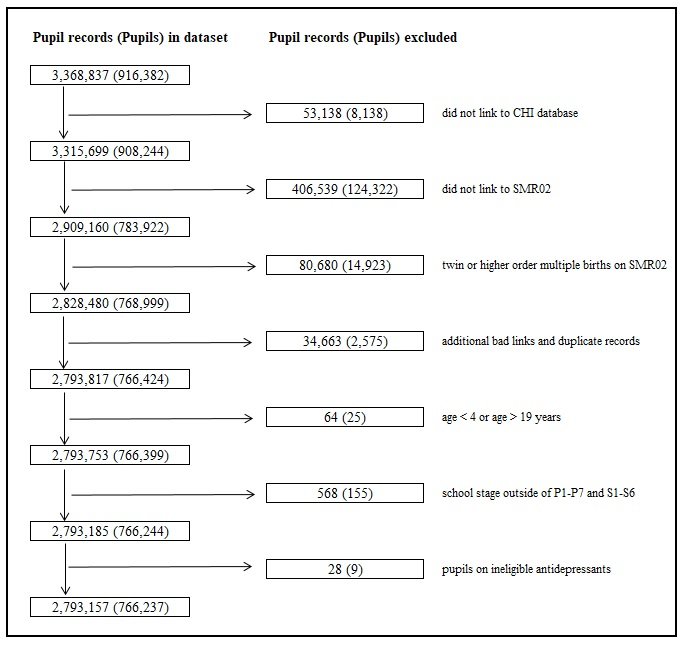

Supplement: dyaa002_Supplementary_Data [file dyaa002_supplementary_data.zip › dyaa002-suppl_data/ije-2018-07-0861-File010.tif]
